# The Medical Man of China

**Published:** 1910-04

**Authors:** John A. Rafter

**Affiliations:** Buffalo, N. Y.


### The Medical Man of China

By JOHN A. RAFTER, M. D., Buffalo, N. Y.

WITH the exception of the treaty ports, and along the western border, the dark curtain of superstition and intolerance still hangs low over China. Within the pale of what we call civilisation, progress and enlightenment, due to contact with western ideas, has made head during the last decades, but in the great interior the people are yet bound together by the peculiar laws, customs, literature, faith, and ceremonies ordained and in practice a thousand years before Christ. Looking backward rather than forward—repellant to advancement,—wanting in enterprise, following in the paths made by its ancestors, indifferent and contemptuous, the Central Empire, so far as the outer world is concerned, has lain dormant for centuries. There is a civilisation in China, but it is the civilisation of long ago. That conditions are now changing and improving, along these lines, and changing rapidly too, cannot be questioned, and the time is not far distant when transformed China will be a factor for all nations to reckon with.

To the western "barbarian" the man of the Flowery Kingdom is something of a triangle, and his reasoning extremely difficult to follow. He has strange and unusual ways of doing things, but after a fashion he accomplishes his object and arrives at results, and sometimes does it well. I have made several visits to China. As an officer in the medical corps of the American army I had a part in the expedition for the relief of the beleaguered legation at Peking at the time of the Boxer uprising. I had been in China before that time, and have but recently returned from the Orient having been absent for more than three years.

As a medical man I was interested in work along that line, and, added to my own observation, I am indebted to medical friends and others who live there as to how the Chinese physician lives, and practises medicine among his people. Excepting those who are located in coast cities, or attached to mission hospitals, there are no educated physicians in China. In the interior of the country there has been no advancement or progress in medical science for the past thousand years. The medical man has no knowledge of medical colleges, and no fear of examination tests before his eyes and is allowed to prey on the folly, ignorance and superstition of the people in a most amazing way. The position of the ruling power on the subject is expressed as follows: "It is the business of the sick to know whether the doctor of their choosing knows his business or not. If they have not brains enough for that, the sooner the world is relieved from their stupidity the better it is for the brains that are left." This sentiment is in effect in China and malpractice suits are unknown. Human nature is alike the world over, and in noting the Chinese physician's opinion of his own ability and his contempt for the opinion of his competitor, I was often reminded of the condition of affairs in the same profession in a more enlightened country far to the westward.

In China, as in all other countries, medical competition is keen, but the Chinese doctor has an advantage over his western brother. No code of ethics disturbs him nor makes him sad. He is a firm believer in that old proverb that "he who tooteth not his own horn, the same shall not be tooted," so it comes about when the medical man finds his preserves being encroached upon, he is much given to making crow's tracks and upside-down-saw bucks on a large and highly colored sheet of paper, which he hangs outside of his office and where all can read. The translation of a few of his advertisements are as follows:

Towers are measured by their shadows, and great men by those who envy them.

I am the seventh son of a seventh son. I am endowed therefore with all the combined wisdom of forty-nine eminent doctors. My charges are moderate.

My feet are planted among the secrets of the earth, and my head is lifted among the discoveries of heaven.

My branches are wide and my roots penetrate deep into the earth. I cannot be overthrown by wind.

I am no blind fowl pecking at random for worms. My knowledge is sure.

I do not climb a tree to hunt for fish. or turn a summersault in an oyster shell, nor am I a toad in a well, contemplating a patch of blue sky, but I survey the universe as from a dome and take in at a glance all real and imaginable things.

In my head are all the maxims of the medical god. All the arts of the imperial leech. All the prescriptions of the philosopher. All the magic the genii unwound from his queue and all the rules of the reckoner.

When I call diseases they answer to their names. Spirits vanish. Principles, elements, and forces assort themselves before me like feathers under the fingers of the flower maker. At my bidding disorders of the most complicated nature resolve themselves into their several members and form action; color and sound have each a tongue to tell me what they mean.

The medicines I dispense are of miraculous virtue and the gratitude of my patients has transformed the garden of my good works into a grove of fragrant almond trees. I apply myself with equal science and concern to all. I skilfully treat the corn on the toe of the mouse catcher or the growth in the eye of the mandarin.

It may be that the Chinese doctor rather overdoes the thing in the way of advertising. yet his statements are in no way more preposterous than those made by advertisers along the same line in this country. and people here are just as easily influenced by such high sounding impossibilities.

In China, physicians do not dissect the human body, and in consequence, scarcely know the position of the greater viscera and the knowledge of the functions and uses of these necessary organs is not very coherent. The theories regarding the anatomical construction of the body are more or less startling. It is thought however that Chinese anatomy differs in a marked degree, and is constructed on a much improved plan to that of other individuals not of the celestial empire. It is told of a Caucasian patient who fell ill, and incidentally into the hands of a native practitioner who was at something of a loss to know what to do for him. a by-stander suggested that acupuncture could do no harm and might help out. "What nonsense, what rashness, exclaimed the doctor. How do you know how these foreign devils are made, and how can you tell what I would be sticking my needle into?" .

Nothing at all is known of the circulation of the blood, but twenty-four pulses are distinguished, one for each organ of the body, and in order to make a correct diagnosis, the condition of each must be ascertained—four or six—according to the experience and dexterity of the physician and counted at the same time. So it is the pulses are felt, much as a rapid performer plays on piano keys. The left pulse is supposed to lead to the heart and the right one to the lungs. The one leading to the spleen is the superior pulse of the body and any disturbance of it indicates a dislocation or disarrangement of the bones of the pancreas. Under certain circumstances depending upon the ruling elements and the relation of the male and female principles of nature, the blood is sometimes light and sometimes dark. The seat of all intellect is located in the east side of the stomach. We dream with the liver and perspire with the lungs.

When called to treat the "son of heaven" or any of his rather numerous family, if the doctor has any desire to retain physical relationship between his head and shoulders it is well for him not to make mistakes, although he would seem to be at a disadvantage. He is required by imperial ukase to remain one rod distant from the august patient. He is not allowed to ask questions nor is he given much time in which to make his long distance examination. If the patient happens to be a lady, the doctor is not allowed to enter the room but is halted in a hall or room some distance from the sick chamber. a string is then attached to the lady's wrist and extended to where the doctor happens to be. He feels the end of the string, gets the condition of the pulse from it, and proceeds to make a diagnosis of the case.

The theory of disease in China is to the effect that a dual system of heat and cold exists in the human body, and if either is in excess sickness results. Now it is the business of the doctor to "strengthen the breath, put down phlegm, repress humors, purge the liver, remove noxious matter, stimulate the gates of life, and restore harmony."

The people have great faith in acupuncture in all rheumatic or painful diseases, and so soon as the physician makes up his mind that some particular bone or muscle is in a state of disorder, he thrusts a substantial needle either cold or red hot into the offending member and stirs it ruthlessly about. He does not hesitate to puncture the abdomen, liver or any other part where inflammation does or does not exist, as the idea is to let out the fire. Blind eyes are punctured for the double purpose of letting out fire and letting in light through the same puncture.

Happily for the people, they are of a lymphatic temperament and do not mind such things.

The Chinese are rather indifferent to smallpox and other contagious diseases and no isolation or quarantine regulations are observed, but they have great fear of typhus and typhoid fever, both of which are very prevalent. Sufferers from such diseases receive but scant attention, as people regard this sickness as due to the presence of devils who are not well disposed, and with which they prefer to have no familiarity. The sick person is placed in a room on the floor and from which all furniture has been removed. A vessel of water is placed near him. The door is secured and a pole laid near it with which, two or three times a day, the anxious relative cautiously peering in, pokes and prods the sick person to discover if he is still alive.

All sorts of substances are used as medicines. Those in daily use and of any remedial value are obtained from native plants. Ginsing is held in great repute. Much that is not only inert but disgusting finds a place in the Chinese materia medica. The tops of medicinal plants are supposed to be good for the cure of diseases of the head and upper part of the body, while the roots are given for diseases of the lower parts. Glue, in some form, such as the gelatinous extract from the long boiled tendons of certain animals, is one of the most commonly used and forms a part of a large number of the combinations given.

The value of medicine is in direct ratio to the disgusting smell and taste it carries with it. Omitting many of the most obnoxious, I will give a few prescriptions, all of which are in use:

*For an Astringent.*—Powder made from dried, ground toads, varnish and glue mixed.

Powder from scorpions mixed with the powder from ground peach stones.

*For a Tonic.*—Extract of bear's jaws and nails. Tiger's bones made into pills. Shavings from stag's horns.

The reasoning for administration of the last one would be: Bears are very strong, and the jaws and nails fierce and strong. Tiger's bones represent the strength of the animal. Stag's horns are also helpful. This patient needs strength.

*To Reduce Fever.*—Powder moths mixed with glue. Rust obtained from old coffin nails. Extracts of insects, especially cockroaches.

*For Cough.*—Earth worms mixed with honey. Glue mixed with oyster shells.

*To Expel Wind.*—Extracts from the feet of monkeys and bats, asbestos, cuttle fish bone and bird nests.

I cannot tell whether children cry for these articles, but they get them just the same.

The medical gods hold a large place in Chinese practice, and all serious cases are called to their attention. This is done by thoroughly rubbing the ears of the god so he may hear, then the messenger rubs his own anatomy at the place where the disease is supposed to exist in the patient. He then rubs the god in the corresponding part after which a highly-colored piece of paper is purchased which seems to have the god's approval. This is taken home, burned and the ashes given to the patient in tea or other hot beverage.

Such are some of the conditions existing at this time in aged sleepy and bad-smelling China. But the leaven is working. Asia is now within the sphere of the rush and whirl of the world's affairs. It cannot go back into darkness. It must go forward into the light. China has many needs and among the most important is an understanding, if such a thing is possible, of that strange peculiar being that helps to make up its four hundred millions of people.

238 Delaware Avenue.

